# Notes and News

**Published:** 1900-09-01

**Authors:** 


### Notes and News.

Our next issue—that, namely, for September 8th—will be our Annual Students' Number, in which, in addition to other matter, subjects of especial interest to those proposing to enter the medical profession will be fully dealt with.

The epidemic of cholera which is raging in India is reported to be one of the worst on record. It is terribly sudden and fatal in its effects, and has already claimed several high officials as its victims, besides attacking several British regiments with severity.

Dr. Collins, F.R.C.S., of the London County Council, has been appointed by the Prince of Wales to be a member of the Council of his Hospital Fund for London. Dr. Collins has served for some time as an honorary secretary of the League of Mercy, where his services have been most valuable. Dr. Collins has done much to stimulate interest in the hospitals through the League of Mercy by addressing large meetings and explaining the aims of the society, and how best they can be furthered.

Livingstone College, which is devoted to the training of medical missionaries, has acquired new premises at Knott's Green, Leyton. Four thousand pounds have been subscribed towards the cost, but another £4,000 is required to complete the work. Dr. Harford Battersby is anxious to obtain the names and addresses of friends and associates of the late Dr. Livingstone, that a representative gathering of those in sympathy with the memorial may be present at the opening of the new college. The office is at 133, Salisbury Square, E.C.

The Company of Drapers have sent to the Prince of Wales's Hospital Fund for London its annual subscription of £1,000. The clerk to the company, Mr. W. P. Sawyer, when forwarding the cheque, writes that "the company learnt with much satisfaction, from the last report of the Prince of Wales's Hospital Fund, that the committee have appointed visiting sub-committees, consisting of persons practically acquainted with hospital management, with the object of obtaining information as to the merits and needs of the various institutions. The company regard this as a most important step towards promoting the well-being of metropolitan medical charities, and strengthening their claims on public benevolence."

Dr. George William Balfour has been appointed a physician-in ordinary to the Queen for Scotland, in place of the late Sir T. Grainger Stewart.

Typhoid fever is again largely prevalent in Paris. The authorities are blamed for their failure to secure a pure water supply for the city.

Lady Rothschild has promised to furnish an isolation hospital at Tring, and to give £300 towards the building.

The following gentlemen are successful candidates for commissions in the Royal Army Medical Corps at the recent examination in London: Mr. H. J. McGrigor, Mr. W. R. P. Goodwin, Mr. J. H. Branskill, Mr. A. C. Duffey, Mr. A. W. Gibson, Mr. C. D. Myles, Mr. R. N. Hunt, Mr. H. E. Howley, and Mr. R. F. M. Fawcett.

In 1898 Mrs. Eliza Roberts left £55,000 to be used for the benefit of the poor of St. Anne's, Soho. The Charity Commissioners have now decided to utilise the money to secure accommodation for the convalescent inhabitants after illness, and to sending the working boys of the parish to seaside camps.

The Hospital Commission is carrying on its work in South Africa with diligence, and a large number of witnesses have been examined as to the condition of the various hospitals, very few complaints being made except as to the difficulty occasioned in some instances by the limited amount of rolling stock, and the excessive red-tapeism.

An extraordinary instance of poisoning has occurred at Rochdale, through which two children have lost their lives, and 150 persons have been rendered ill. The cause is understood to be some ice cream partaken of at a Sunday-school fête. The vendor was exonerated from blame, there being nothing to show carelessness or the introduction of any deleterious matter save by accident.

A commission has been appointed to inquire into the cause and prevention of dysentery in the field. The commission, consisting of Dr. Notter, of Netley Hospital; Dr. Simpson, of King's College; and Major Bruce, R.A.M.C., is especially charged to trace any connection which it is possible to find between dysentery and typhoid.

In Melbourne a method of securing patients' payments has been adopted which has proved very successful. Patients are required to sign a declaration as to their earnings, and when able to do so they are asked to contribute according to their means. The payments have varied from a few shillings to thirty shillings a week, and the income of the hospital has increased by £3,000.

The metropolitan medical schools open for the most part on October 1st. On the evening of that day the annual dinners of the following hospitals will take place: University College, at the Trocadero Restaurant; St. George's Hospital, at the Hotel Metropole; St. Thomas's Hospital, at the Hotel Metropole; King's College Hospital, at the Hotel Cecil; the London Hospital, in the College Library; the Westminster Hospital, at the Café Monico. On October 2nd the dinner of the Charing Cross Hospital will be held at the Hotel Cecil; on October 3rd that of St. Mary's Hospital, at the Hotel Metropole.

